# Bees, flowers and UV

**DOI:** 10.1111/plb.70050

**Published:** 2025-05-22

**Authors:** K. Lunau, M. G. G. Camargo, Z.‐X. Ren

**Affiliations:** ^1^ Faculty of Mathematics and Natural Sciences, Heinrich Heine University Düsseldorf, Institute of Sensory Ecology Düsseldorf Germany; ^2^ Department of Biodiversity, Center for Research on Biodiversity Dynamics and Climate Change, Phenology Lab Biosciences Institute, UNESP–São Paulo State University São Paulo Brazil; ^3^ CAS Key Laboratory of Plant Diversity and Biogeography of East Asia Kunming Institute of Botany, Chinese Academy of Sciences Kunming Yunnan China

**Keywords:** Bee vision, flower colour, fluorescence, glossiness, reflectance, UV pattern, UV radiation

## Abstract

Ultraviolet light shining on flowers has various effects. In this review we assess functions of UV pigments and UV reflection patterns in flowers, including visual signalling by reflectance, fluorescence, and gloss, as well as protection against UV radiation. UV patterns originate from UV reflection and absorption in different floral parts and are visible to most pollinators, but invisible to humans. UV patterns can guide pollinators towards a floral reward, such as the centre‐outward UV pattern, the so‐called UV bull's eye. However, the diversity and complexity of floral colour patterns is much higher and may or may not include UV. For flower visitors, reflected UV light is merely a component of their colour vision rather than a UV signal processed separately. Yet, to humans it is a challenge to detect and represent UV reflectance in flowers. Advantages and limits of spectrophotometry, UV photography and false colour photography in bee view are discussed. Besides floral pigments causing absorption and fluorescence, flower signals can be produced by epidermal structures, i.e. smooth or conical epidermal cells, causing specular reflection (gloss) or refraction of light, and light‐scattering structures causing reflection. Exposed nectar, pollen and stamens also display visual signals including UV. Finally, the absorption of UV light by pollen pigments protects the precious DNA inside the pollen grain from harmful UV radiation. UV‐absorbing central parts on flowers also protect flower DNA by impeding the reflection of UV light from petals onto stamens and pollen. We briefly discuss how flower UV patterns may change in response to increasing global UV radiation, potentially influencing plant pollination.

## Graphical abstract

Human‐invisible but bee‐visible UV patterns in flowers aid pollination by signalling to pollinators and protecting vulnerable DNA in pollen.

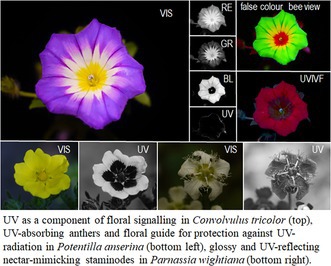

## INTRODUCTION

**Fig. 1:**
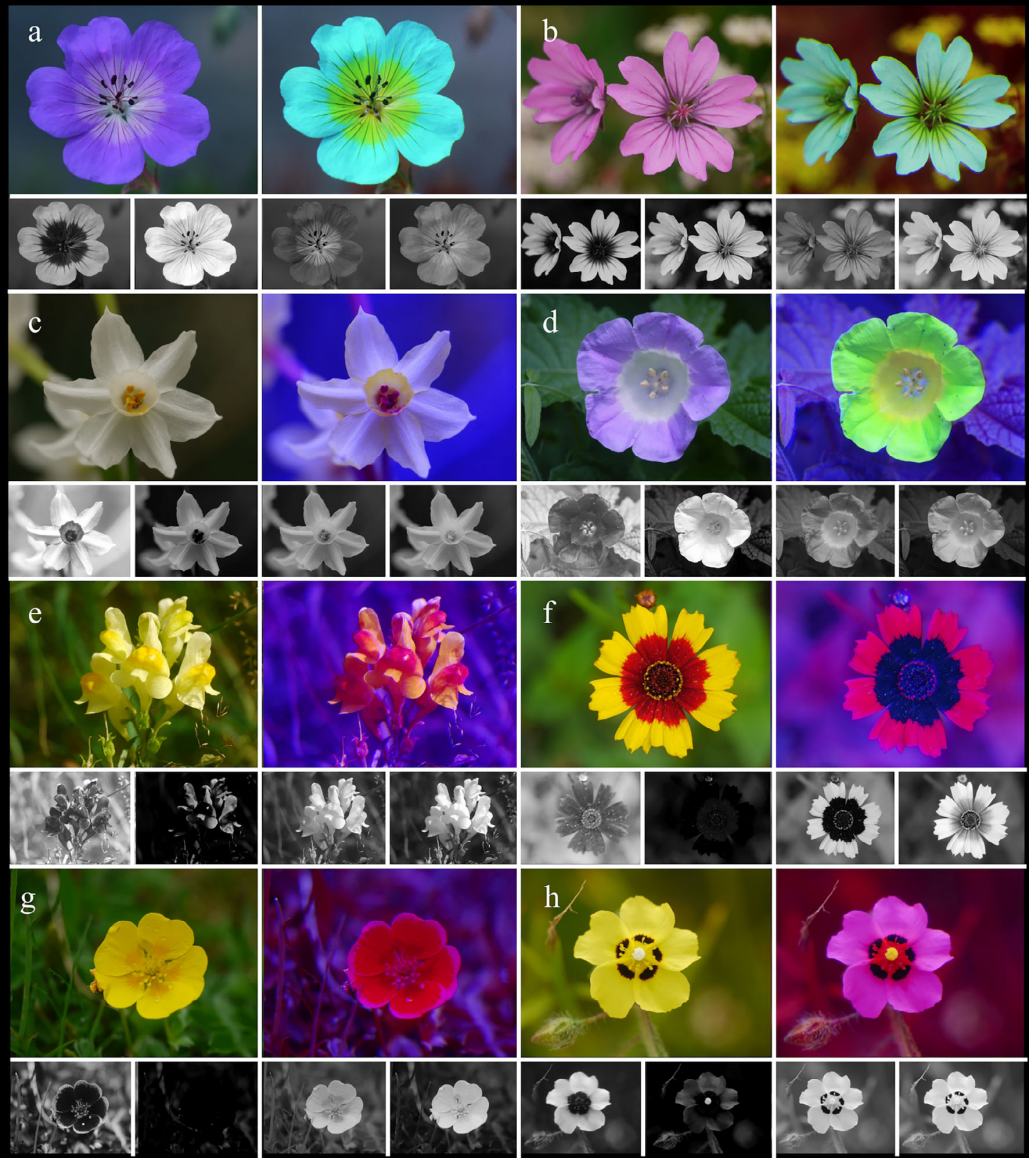
Diversity of UV patterns with colour photo (top left), false colour photo in bee view (top right), blue channel of the UV photo, blue, green, and red channel of the colour photo (bottom from left) of (a) *Geranium sanguineum*, (b) *Geranium pyrenaicum*, (c) *Narcissus papyraceus*, (d) *Nicandra physalodes*, (e) *Linaria vulgaris*, (f) *Coreopsis tinctoria*, (g) *Potentilla aurea*, and (h) *Tuberaria guttata*.

Most flower visitors, including bats (Domingos‐Melo *et al*. 2021), birds, butterflies, hawkmoths, hoverflies and bees, possess the remarkable ability to perceive ultraviolet (UV) light—a sensory capability that eludes human vision (Lunau & Maier 1995). This UV sensitivity enables pollinators to detect intricate signals on flowers, such as UV patterns, which remain invisible to us. A colour pattern is defined as any pattern composed of two or more colours within the same attractive structure, be it a flower, an inflorescence, or any other signalling apparatus relating to plant pollination, no matter the plant organs involved (Lunau *et al*. 2024). Consequently, a UV pattern is a colour pattern restricted to differences in the reflection of ultraviolet light (Fig. 1), although a UV pattern can be associated with a visible colour pattern.

Flower colours are a function of pigments that absorb light quanta over a particular range of wavelengths. For example, blue flowers possess blue pigments that absorb green and red light, whereas backscattered blue light causes the blue reflection (van der Kooi *et al*. 2019a). Some confusion might arise due to the meaning of the term “pigment”: A yellow pigment, such as a carotenoid, per definition absorbs blue light and thus enables the backscattering of green and red (perceived as yellow) light. However, the term “UV pigment” is sometimes used to describe a pigment that absorbs ultraviolet light (see Gronquist *et al*. 2001; Moyers *et al*. 2017). Typical flower pigments, such as carotenoids, flavonoids, aurones‐chalcones, cyanidins, pelargonidins, delphinidins, and malvidins, vary in their UV‐absorbing properties (Narbona *et al*. 2021). Pigments that have absorbed light quanta can cause the re‐emission of heat or fluorescent light. UV‐induced visual fluorescence causes an increase in emitted visual light, a decrease in UV light penetration further through the floral tissue, and a decrease in heat production. Fluorescence thus potentially affects the visible light emitted from flowers as well as rendering UV radiation less harmful (Van der Kooi *et al*. 2019b).

Besides light‐absorbing pigments, structures on the petal surface can modify the reflected light or cause total reflection. Conical epidermal cells function as light traps and can focus the incoming light onto the pigment layer and thus modify the visual colour signal (Gorton & Vogelmann 1996). Smooth epidermises cause total angle‐dependent reflection of light including all visible wavelengths and UV, that is, gloss, which might be a strong visual signal (Van der Kooi *et al*. 2017). Gloss is also caused by smooth structures such as exposed nectar. The structure of the petal epidermis can also affect heat absorbance, grip for pollinators, and wettability (Papiorek *et al*. 2014).

Colour is often synonymized with hue, but hue is only one aspect of colour. Colour offers more features than hue, such as brightness, saturation, and contrast to the surrounding colour (Lunau & Dyer 2024). The frequency of flower colours in distinct habitats has provoked hypotheses about honest signalling by flowers and colour preferences of pollinators. It might be appealing to conclude from the frequent visits to blue flowering species by bumblebees that bumblebees either possess a preference for blue colour (Dyer *et al*. 2021), or that the blue flower colour honestly signals a higher nectar reward (Streinzer *et al*. 2021). However, there is no evidence that bumblebees innately prefer blue colours (Lunau & Dyer 2024) nor do blue flowers offer more nectar (Shrestha *et al*. 2020; Streinzer *et al*. 2021; but see Giurfa *et al*. 1995).

Probably because of the diversity and complexity of floral colour patterns (Fig. 1), and the manifold and different ways to demonstrate how flower colours are viewed by bees, there is uncertainty, or sometimes even misdirection, in the popular scientific and even in the scientific literature about how bees see flowers. As an example, although researchers know that UV is merely a component of colour vision, they cannot resist the temptation to emphasize UV as a discrete signal. Ultraviolet, and particularly UV patterns on flowers have been considered as a special signal for pollinators: Klomberg *et al*. (2019): “…effect of UV signalling on attracting pollinators”; McCarren *et al*. (2021): “pollinator preferences for UV reflectance”; Turatbekova *et al*. (2023): “…ultraviolet (UV) reflectance serves as a significant visual advertisement for pollinators…”; Lunau *et al*. (2021): “…the exaggerated UV signal is strategically relevant in floral mimicry…”. The common term “UV signal” is somehow misleading, since it suggests that the reflection of ultraviolet light produces the entire signal. However, the ultraviolet bull's eye, the most prominent UV signal, absorbs UV light. The term “UV signal” might even misdirect researchers to assume that bees perceive only ultraviolet light or process exclusively ultraviolet information. The importance of UV as a component of flower reflection has often been exaggerated, but Kevan *et al*. (2001) clarified that ultraviolet is merely one component of visual stimuli for UV‐sensitive animals.

In practice, the most frequently asked questions and the key issues requiring clarification are:

- Are there flowers that are bee‐UV (Shrestha *et al*. 2014)?
- Are there flowers or parts of flowers that are invisible to bees (Chittka & Waser 1997)?
- Many flowers display the so‐called UV bull's eye; what is it and are there also blue and green bull's eyes (Lunau 2014)?
- How do bees respond to flowers lacking a UV pattern?
- How do bees perceive different features of flower colours, such as colour contrast, brightness, hue, and saturation?
- Is UV a specific floral signal?
- Does fluorescence contribute to flower colour?
- Is UV an important component of glossy flower signals?
- Do flowers more often display UV‐absorbing parts in high UV radiation environments?
- Are distinct features of UV patterns associated with altitude and/or latitude?

The aim of this review is to summarize our current understanding on flowers, bees and UV. We assess biotic functions of UV pigments and UV patterns in flowers, such as visual signalling to bees, causing autofluorescence, and simulating gloss, as well as protection against UV radiation. We try to clarify and answer the questions listed above and define the knowledge gaps for directing research in the future.

### UV sensitivity of bee pollinators

The bees' sensitivity to UV light has puzzled pollination biologists for decades (Daumer 1958; Silberglied 1979; Kevan 1983; Biedinger & Barthlott 1993; Chittka *et al*. 1994; Kevan *et al*. 2001; Papiorek *et al*. 2016) and is still a matter of discussion (Chen *et al*. 2020; Tunes *et al*. 2021). The difference in sensitivity to UV light in humans and most pollinators fuels the search for “private” messages from flowers to their pollinators that humans cannot see.

Colour vision in humans and in bees differs in the relevant sensitivity range of wavelength; bees are sensitive to UV light, humans are not, whereas humans are more sensitive to red light compared to bees (Lunau & Maier 1995). However, we should keep in mind that humans and bees share sensitivity to blue and green light. Thus, to understand flower colours as seen by bees requires a “translation” from bee colours to human colours. The UV gap in human colour vision and the red gap in bee colour vision have dramatic consequences. For example, red bee‐ and red bird‐pollinated flowers differ in the reflectance of UV light (Lunau *et al*. 2011). Most red bee‐pollinated flowers reflect UV light and thus appear bee‐UV to bees. In contrast, red bird‐pollinated flowers are often UV‐absorbing and thus appear bee‐black, which might discourage bees from visiting these flowers. Usually, bees cannot pollinate red bird‐pollinated flowers, they merely exploit them as nectar thieves or pollen robbers (Lunau *et al*. 2011).

However, there is often too much focus on the UV aspect of flowers. UV photos are often shown (Fig. 1), while blue or green photos are not (Lunau *et al*. 2024). UV photos of flowers displaying a UV pattern are published frequently but one rarely sees photos of completely UV‐absorbing flowers although they are common (Biedinger & Barthlott 1993). UV is often treated as an extraordinary feature of flowers; for example, the term ‘UV‐bull's eye’—a term coined by Silberglied (1979) indicating a UV‐absorbing centre of a UV‐reflecting flower—is much more often highlighted than blue or green bull's eyes (Lunau *et al*. 2024).

**Fig. 2:**
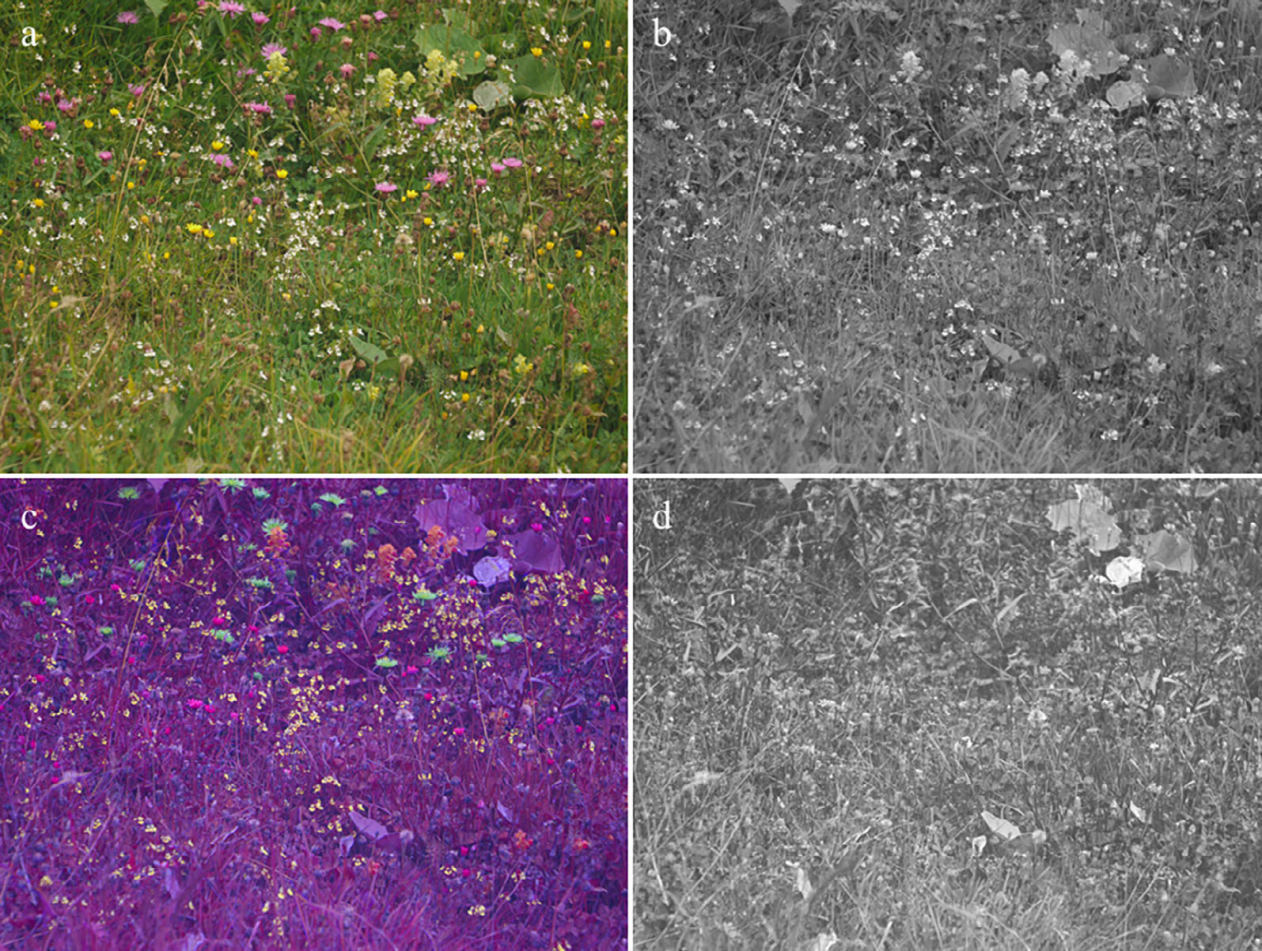
View of a meadow. (a) Colour photo, (b) green contrast photo, (c) false colour photo in bee view, and (d) UV photo of an alpine meadow on in the Beverin Natural Park, Switzerland.

Ultraviolet‐sensitive pollinators can detect intricate signals on flowers, such as UV patterns, which remain invisible to us. Among these flower visitors, bees, especially commercial honeybees and bumblebees, have been studied extensively regarding their visual orientation. Historically, these studies provided evidence for bee colour vision (Von Frisch 1914), trichromacy (Daumer 1956), colour discrimination (Von Helversen 1972), colour constancy (Neumeyer 1981), colour vision modelling (Chittka 1992), innate colour preferences (Giurfa *et al*. 1995; Lunau *et al*. 1996; Lunau & Dyer 2024), and colour‐blind orientation via green contrast at a greater distance from the flowers (Dyer *et al*. 2008; Hempel de Ibarra *et al*. 2015; Fig. 2). Naïve bumblebees easily detect flowers that contrast against the background, preferably approach flowers of high spectral purity (Rohde *et al*. 2013) and, on flowers displaying a colour pattern, preferably target and land on floral guides displaying stronger spectral purity compared to the periphery, irrespective of hue (Lunau & Dyer 2024). In contrast, naive *Eristalis tenax* hoverflies prefer to approach yellow flowers and innately extend their proboscis only towards yellow (green‐reflecting) and UV‐absorbing areas (Lunau 2014), a fine‐tuned visual response to the colour of pollen (An *et al*. 2018). Insects distinguish yellow UV‐patterned and white UV‐patternless flowers of *Anemone palmata* resulting in partitioning of pollinator groups between the two colour morphs and a marked constancy to flower colour during foraging. While bees and hoverflies are more likely to visit UV‐patterned flowers, other flies and beetles show no preference between UV‐patterned flowers and UV‐patternless flowers (Rodríguez‐Castañeda *et al*. 2024). Moreover, Neotropical yellow bee‐pollinated flowers display a UV pattern, but hummingbird‐pollinated yellow flowers display a uniformly UV‐absorbing colour (Papiorek *et al*. 2016).

### Visualizing UV

For the reasons mentioned above, it is very important to visualize UV for humans to better understand flower colours. For years, scientists have grappled with describing and visualizing flower colours as perceived by bees. To investigate flower colours objectively, researchers employ quantitative methods, such as reflectance spectrophotometry (see below). By measuring the spectral reflectance of flowers, scientists construct reflectance curves that reveal the percentage of light reflected compared to white (100%) and black (0%) standards. The spectral reflectance curves might lead one to believe that flower colour is an inherent property of the flower itself. However, a more nuanced perspective emerges when we consider that flower colours exist in the eye of the beholder—specifically, the bee's eye. Bee photoreceptors are supposed to adapt to ambient light conditions. The current colour vision model for bees (Chittka 1992) assumes that the bee photoreceptors are half maximally sensitive when looking at the background colour (Bukovac *et al*. 2017). Therefore, bee eyes are tuned to detect colours contrasting against the background.

Spectrophotometry allows the quantitative measurement of flower colours, which is required for the calculation of colour contrast and other colour parameters. Many details of flowers, such as stamens, pollen grains, anthers, and floral guides, are too small or insufficiently smooth for spectrophotometry. UV photography allows a semi‐quantitative (Biedinger & Barthlott 1993) or even quantitative (Garcia *et al*. 2014) representation of the UV reflection of these structures. UV photos are taken mostly by modified UV‐sensitive cameras equipped with a UV‐permeable objective and a UV filter that transmits only in the ultraviolet range of wavelengths (Daumer 1958; Silberglied 1979; Verhoeven *et al*. 2018).

**Fig. 3:**
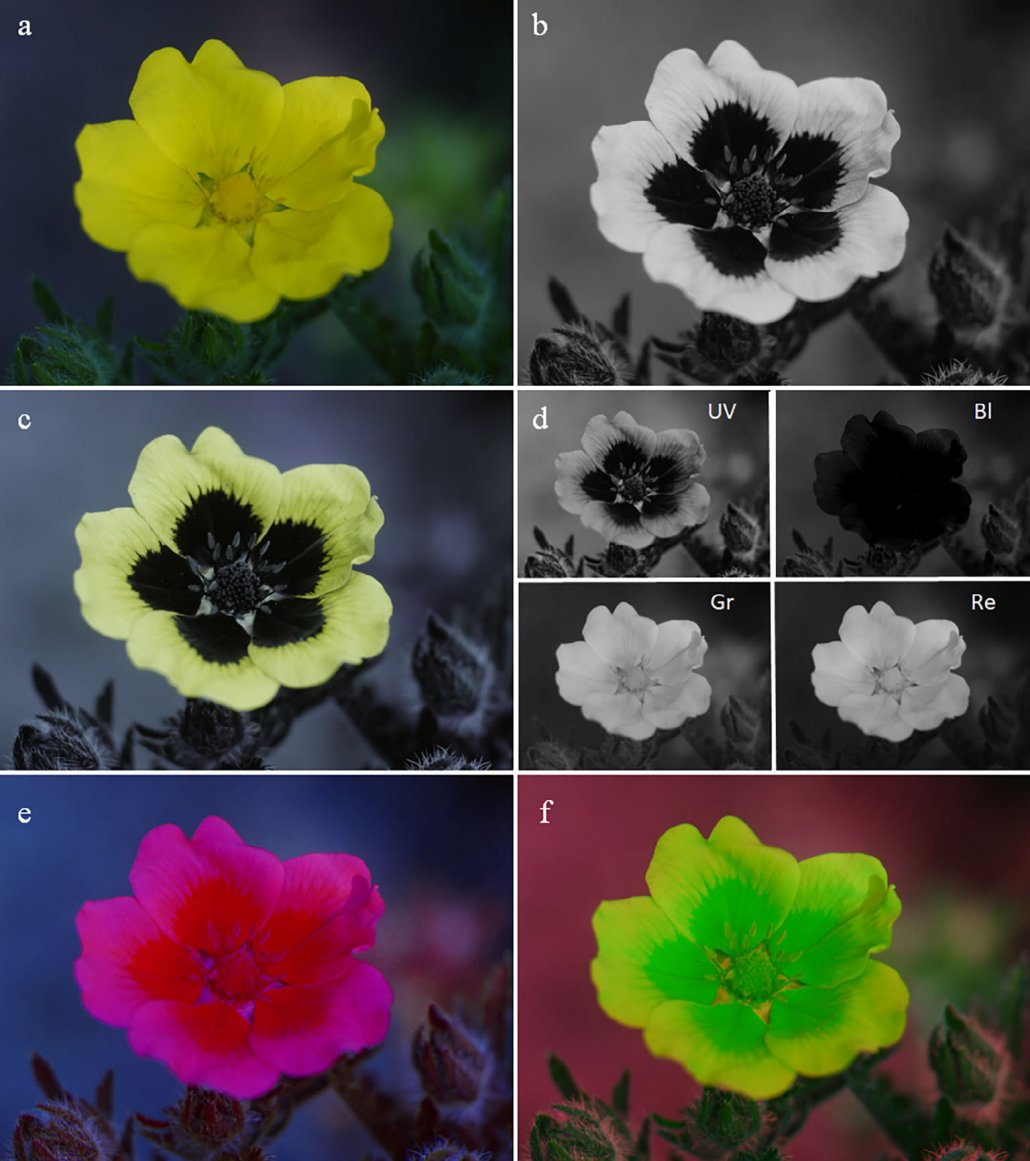
UV bull's eye on a flower of *Potentilla anserina*. (a) Colour photo, (b) UV photo taken with a digital camera as grey‐scale, (c) same photo not modified to grey‐scale, and (d) blue (UV) channel of the UV photo, blue (Bl), green (Gr), and red (Re) channel of the colour photo, (e) false colour photo in bee view with UV as blue, blue as green and green as red, (f) false colour photo in bee view with UV as red, blue as blue and green as green.

The presentation of UV photos varies. Grey‐scale UV photos are presented next to colour photos of the same flower and encourage the reader to superimpose visible colour and UV pattern, which is quite easy if the visible colour is uniform (Fig. 3). Alternatively, UV photos are shown as colour photos, tinted blueish, greenish, or reddish (Fig. 3), caused by the respective channel that is most sensitive in the UV. The tint of UV photos can hamper our understanding. A further difficulty is the fact that from the combination of a colour photo and a UV photo the interpretation of flower colours, as viewed by bees, remains limited because the red range of wavelengths is not excluded. Red is part of human‐visible purple, red, yellow and white colours. False colour photos in bee view resolve this problem.

**Fig. 4:**
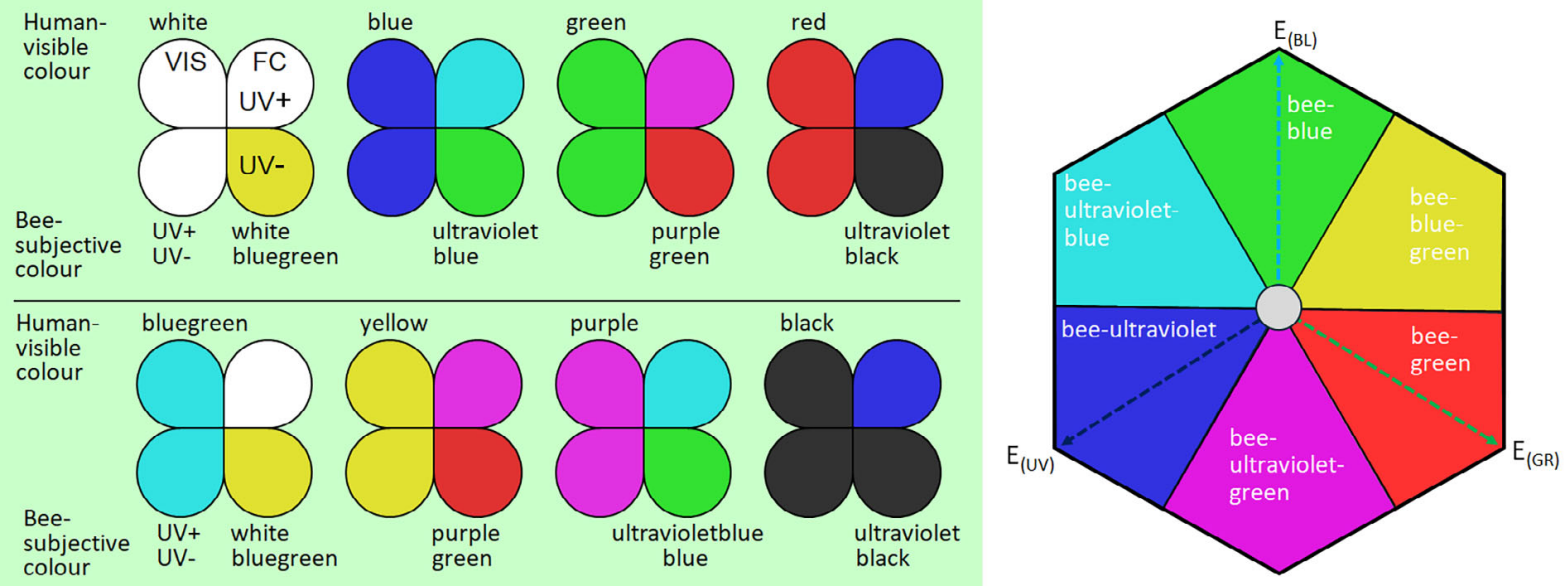
Colour coding scheme indicating human‐visible colours and their names, bee‐subjective colours and their names according to the colour hexagon with and without reflection of UV light. VIS, colour in visible light; FC, false colour in bee view either with (UV+) or without reflection in the ultraviolet range of wavelength (UV−). The colour hexagon (right) shows the plotting of the excitation (E) of the photoreceptors, the bee‐subjective hue categories as in false colour photos in bee view and the uncoloured area in the centre.

The false colour photo is merged by the blue and green channels of a colour photo and one informative channel (depending on camera type) of a UV photo, using a (bathochromic) shift to longer wavelengths, by which UV is shown as blue, blue as green, green as red and red is discarded (Verhoeven *et al*. 2018; Fig. 4). These false colour photos of a bee's view are produced by image editing software on a computer. The result is strikingly similar to hand‐made paintings based on spectral reflectance data of flower colours (Kevan 1978). However, other false colour representations are possible, especially if blue is represented as blue, green as green, while UV is represented as red (Fig. 3).

### Fluorescent flowers

Autofluorescence is a common phenomenon of pigment molecules (Donaldson 2020; Croce 2021). Light is absorbed in a particular narrow range of wavelengths by a fluorophore and subsequently emitted as light of a longer wavelength. Many floral pigments and chlorophyll are fluorescent (Gandía‐Herrero *et al*. 2005). The absorption of light energy by a flower pigment results in an electronic excited state, which can undergo different decay pathways such as heat dissipation, chemical reactions, or light emission as fluorescence, among others. Hypothetically, flowers might benefit in two different ways from fluorescent pigments. First, the fluorescent light emission might add to the reflection of light in the visible range of wavelengths; for example, if a blue flower has UV‐induced fluorescence in the blue range of wavelengths (Marshall & Johnsen 2017). Second, since heat dissipation and fluorescence are competitive processes, an increase of fluorescence can decrease the fraction of energy dissipated as heat. This might also protect anther tissues from overheating and subsequent damage to pollen grains (Mori *et al*. 2018). Autofluorescence of flowers has been particularly found in pollen grains and anthers. The consensus of various studies on autofluorescence of pollen seems to be that sporopollenin is the UV‐excited blue fluorophore, while green/yellow fluorescence may be caused by flavonoids, carotenoids, or lipofuscin, reflecting the complex mixture of potential fluorophores of pollen (Donaldson 2020).

A visual function of fluorescence has been claimed for the presence of fluorescent nectar (Thorp *et al*. 1975; Magner *et al*. 2024; Zenchyzen *et al*. 2024), but this has been questioned as nectar sugars do not fluoresce. In fact, as nutrient molecules in nectar ingredients are at such low concentrations, the sparkling of openly presented nectar would superimpose any colour imparted through fluorescence (Kevan 1976). A recent study on non‐flying, mammal‐pollinated flowers demonstrated blue‐fluorescent nectar, but found no evidence for a role of fluorescence in the signalling to pollinators (Wester & Brühn 2024). Since the quantum yield of flower pigments in petals, that is, the number of emitted photons compared to the number of absorbed photons, amounts at most to 0.015, the fluorescence emission is negligible compared to the reflected light effect. Thus, fluorescence may not be considered as an optical signal in biocommunication between flowers and pollinators (Iriel & Lagorio 2010; Lagorio *et al*. 2015).

The difficulties in finding evidence for the relevance of fluorescent colours in experimental settings are demonstrated in the study of Mori *et al*. (2018). Honeybees spontaneously prefer artificial flowers made of white filter paper soaked in blue fluorescent chlorogenic acid over untreated control artificial flowers. The spontaneous preference of honeybees for these fluorescent artificial flowers was impeded strongly by reduced solar UV intensity caused by cloudiness and by the quenching of fluorescence, that is, the inner filter effect at higher concentrations of chlorogenic acid (Mori *et al*. 2018). Moreover, honeybees' preference for these fluorescent artificial flowers could be caused either by the enhanced absorption of UV light or by the emission of blue fluorescent light (Mori *et al*. 2018). Direct evidence that flower visitors respond to the fluorescence of natural flowers is still lacking.

Fluorescence is used as a tool in scientific studies. Green fluorescent protein is used as a protein tag in flowers. A comparative spectrofluorimetric study shows that the transparency of the petals and cuticle to the exciting UV light is a necessary precondition to observe the green fluorescence (Mercuri *et al*. 2001).

**Fig. 5:**
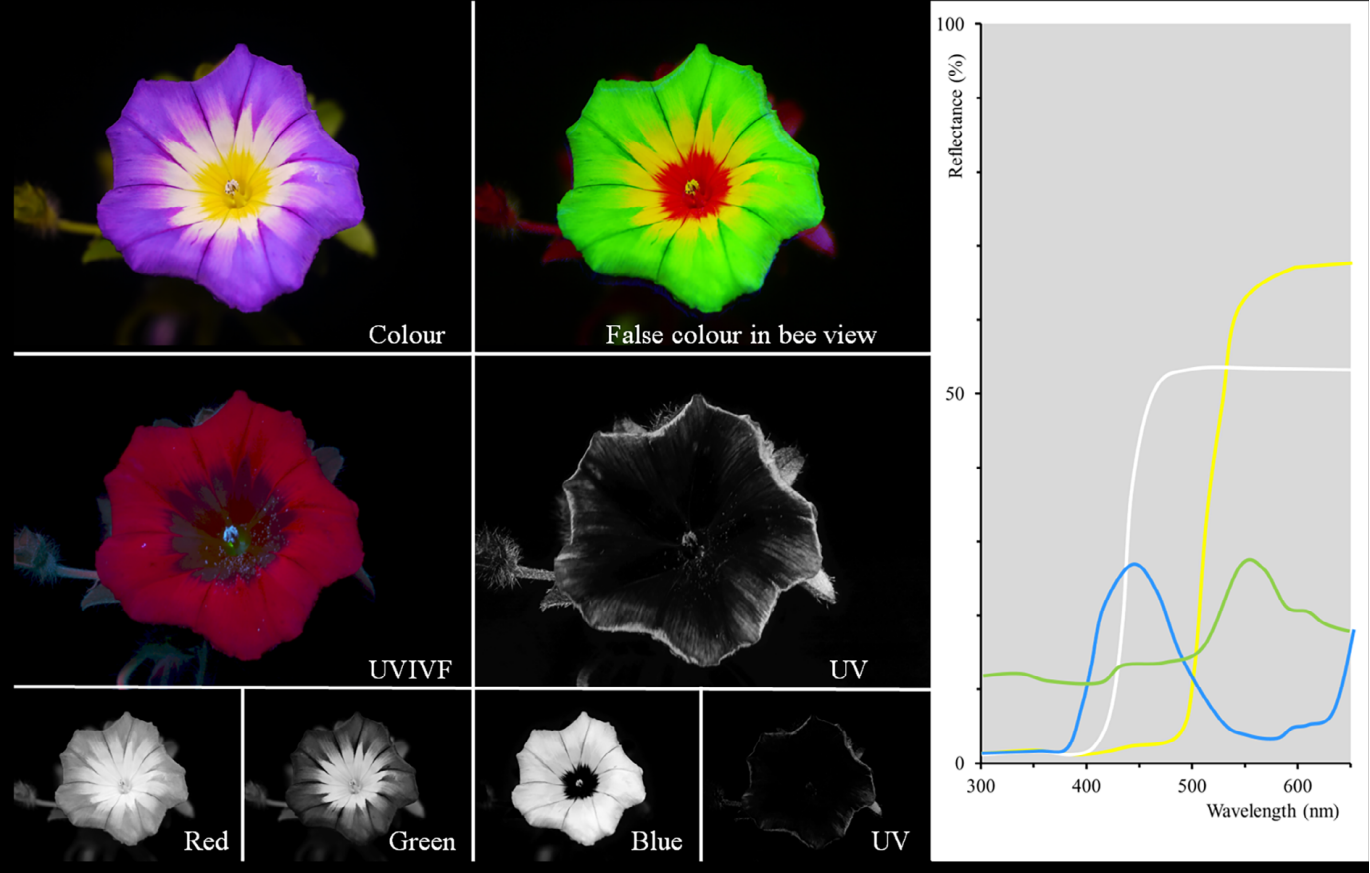
Views of dwarf morning glory (*Convolvulus tricolour*): colour photo, false colour photo in bee view (see text), UV induced visual fluorescence (UVIVF), grey‐scale UV photo and red, green, and blue channel of the colour photo as well as the blue channel of the UV photo used for merging false colour photo in bee view. The spectral reflectance for the green leaf and three petal areas is indicated by their colour.

**Fig. 6:**
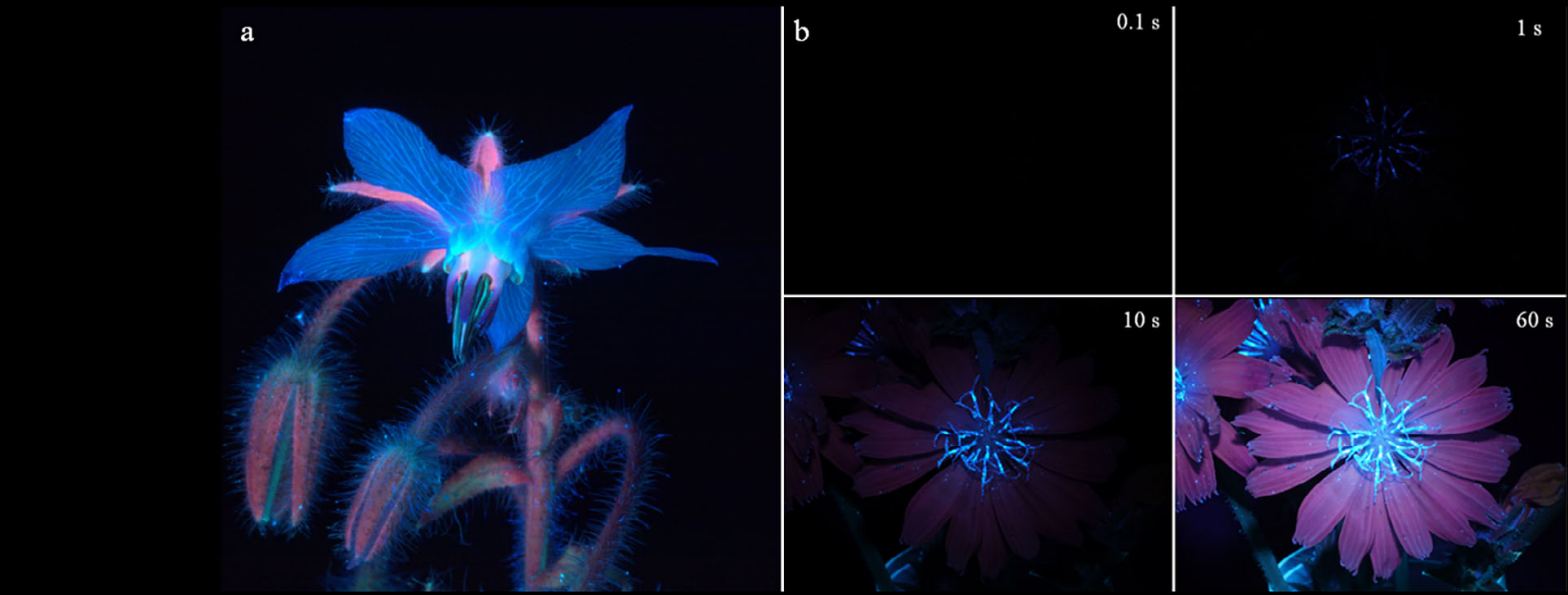
UVIVF photos of (a) Common borage (*Borago officinalis*) and (b) Common chicory (*Cichorium intybus*) taken in the dark with an aperture of 22, but with varying exposure times.

Fluorescent flowers, most of which are highly aesthetic and luminescent, are omnipresent in popular scientific media. Ultraviolet‐induced visible fluorescence (UVIVF) highlights floral fluorescence by illuminating flowers with UV light in darkness and taking a photo within the visible wavelength range (Yearsley 2022). A popular science book (Burrows 2024) presents UVIVF photos of flowers (Figs 5 and 6) under the title “What the bees see.” This publication claims that the UV‐induced visible fluorescence photos demonstrate aspects of bee vision. However, this is erroneous for several reasons. The UV‐induced visible fluorescence photos were taken in the dark, with flowers illuminated exclusively with UV light using an over‐long exposure time under artificial conditions where bees are not active. The amount of emitted fluorescent light under UV illumination is very small, requiring exposure times longer by a factor of >100 compared to colour photos in sunlight (Fig. 6). UVIVF photos do not show UV reflection of flowers that bees can see, ignore visual fluorescence elicited by blue light, but include fluorescence of red light that is invisible to bees. If there is a visible effect of UVIVF, then humans should be able to see the difference between flowers in sunlight and in flowers under indoor light conditions, excluding UV light. However, since the emitted fluorescent light is in the human‐visible range of wavelengths, it should be visible to both bees and humans, but based on our experience, the fluorescence of flowers is invisible to humans when presented in sunlight.

### Fluorescent pan trapping bees

**Fig. 7:**
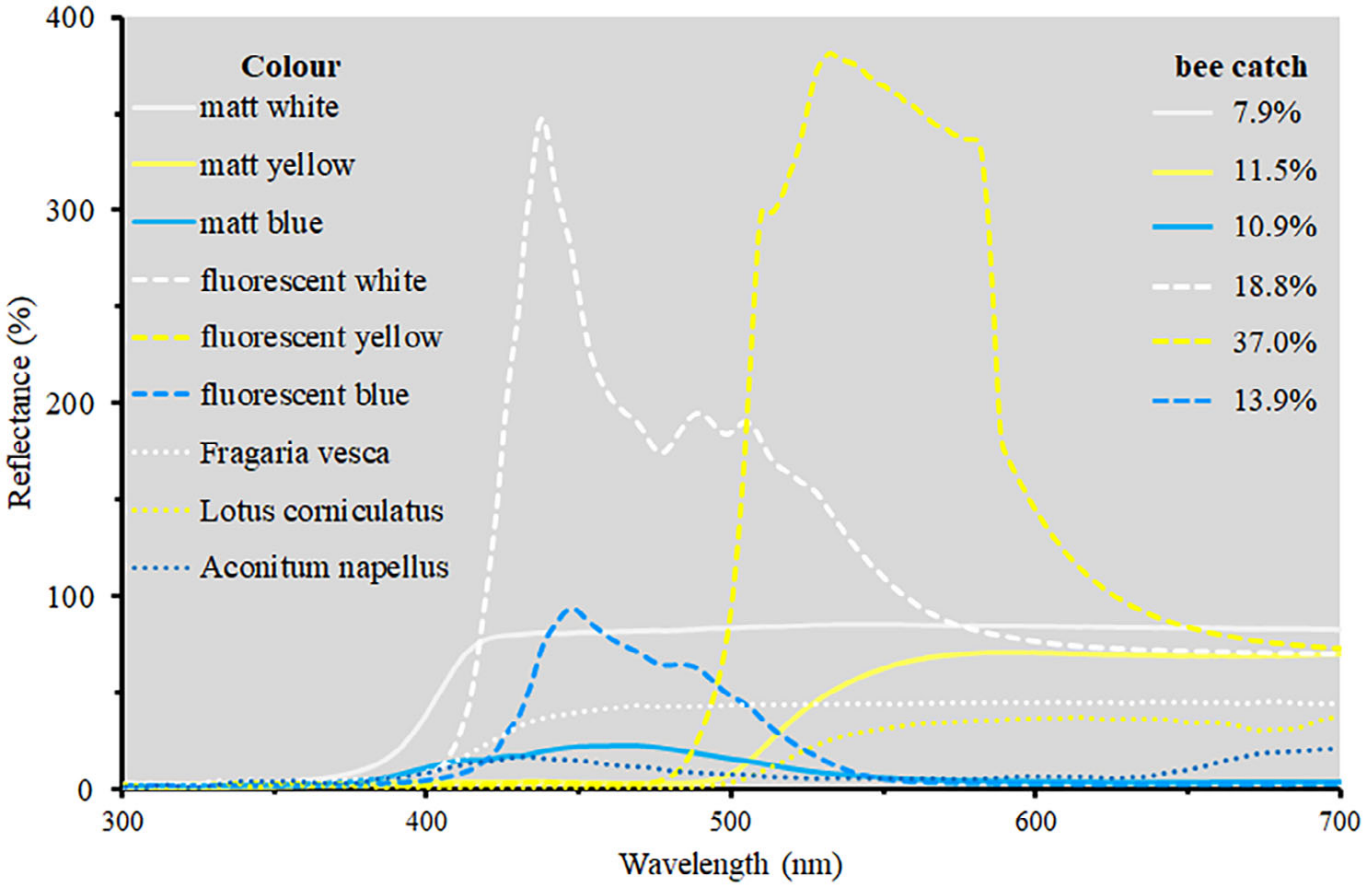
Spectral reflectance and matt (RAL 9010 Reinweiß, RAL 1021 Rapsgelb, RAL 5010 Enzianblau) and fluorescent pan trap colours (Spray‐Colour, Merzenich, Germany: Spavar3108 Leuchtweiß, Spavar 3104 Leuchtgelb, Spavar 3107 Leuchtblau) measured with USB 4000 (OceanOptics, Ostfildern, Germany). Percent bee catch is indicated. Modified after Diestelhorst *et al*. (2014). Spectral reflectance of flowers from Arnold *et al*. (2010).

Artificial colours matching natural flower colours as close as possible are used in experiments with artificial flowers to test innate colour preferences or colour discrimination in bees (Bukovac *et al*. 2016; Lunau & Dyer 2024). Coloured pan traps are also used to catch bees in the wild without sampling bias (Klaus *et al*. 2024). Standardizing bee sampling with pan trapping is important for comparing associated floral surveys (Krahner *et al*. 2024a). In principle, there are two options: pan traps matching the flower colours in the natural habitats and pan traps catching as much bees as possible. Since the diversity of natural flower colours is immense and the UV reflection properties of most paints are neither known nor manipulable, a set of white, yellow and blue fluorescent pan traps seem to be the most feasible option (Krahner *et al*. 2024b). Evidence that bees respond to strongly fluorescent colours arise from pan trap catches, demonstrating the high attractiveness of fluorescent compared to non‐fluorescent colours (Rao & Ostroverkhova 2015; Shrestha *et al*. 2019); in these cases, the fluorescence of colours was visible to the naked eye and resulted in reflectance values up to 350% (Shrestha *et al*. 2019). A comparative study shows that fluorescent pan trap colours attract more bees than similar but non‐fluorescent pan trap colours (Diestelhorst *et al*. 2014; Fig. 7).

### The UV pattern and bee ultraviolet flowers

Patterns of UV on flowers clearly indicate that UV‐absorbing and UV‐reflecting colours are perceived as different colours by bees (Figs 1, 3 and 4; Lunau *et al*. 2024). In general, flowers are predominantly UV‐absorbing and colour patterned, and UV patterns are expected to be less common than patterns promoted by other colours (Chittka *et al*. 1994; Heuschen *et al*. 2005; Tunes *et al*. 2021). However, UV patterns are strongly associated with bee pollination and are an important visual cue for bees (Papiorek *et al*. 2016; Camargo *et al*. 2019). The response of naïve and untrained bees to colour pattern has been tested with model flowers. Bees do not spontaneously direct their approach towards the centre of flowers. The display of colour patterns directs the bees' approach and first contact with the flower to the floral guide (Lunau *et al*. 2006). The crucial parameter is not the colour contrast between the floral guide and the main flower colour, although a certain colour contrast is essential. Bees are attracted towards the floral guide, if the floral guide colour is more saturated than the main flower colour (Lunau & Dyer 2024). That means, testing the bees' response to reciprocally coloured model flowers and floral guides with identical colour contrast will not result in a similar response. Rather, bees approach the model flower with the more saturated main colour, but will direct their approach more often towards the floral guide of the less saturated model flower that displays the more saturated floral guide.

**Fig. 8:**
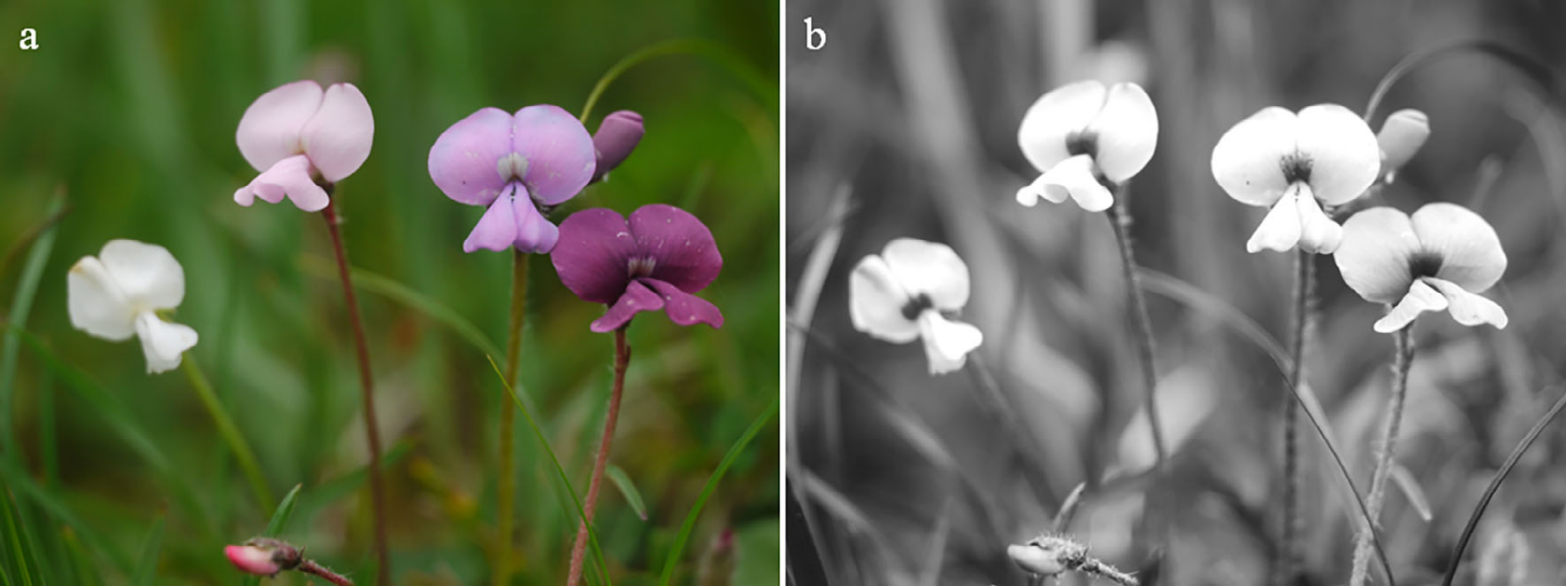
(a) Colour photo and (b) UV photo of different colour morphs of *Tibetia yunnanensis* on Yulong Snow Mountain, Yunnan, China.

Generally, patterns of flowers mostly consist of a UV‐absorbing centre and a differently coloured periphery, being UV‐reflective or not (Fig. 1). Even white colour morphs of flowers often retain the UV pattern, indicating that UV absorption is caused by additional pigments (Lunau 2022; Fig. 8). In some cases the colour pattern has more than two components, for example additional dark lines or dots, as in *Tuberaria guttata* (Fig. 1h), or central UV‐absorbing parts with more than a single colour, as in *Potentilla aurea* (Fig. 1g). Blue, yellow, red, and purple flowers are common. Green flowers are rare due to their lack of conspicuousness against green vegetation (del Valle *et al*. 2024). Bee‐pollinated white flowers strongly absorb UV light and thus are bee‐bluegreen (Figs 1, 4, and 7) for several hypothetical reasons. Therefore, UV‐reflecting white flowers provide little colour contrast against a background of green leaves (Kevan *et al*. 1996). UV‐reflecting white flowers are less saturated for bees compared to UV‐absorbing white ones and thus they fail to match the bees' innate flower colour preference (Lunau *et al*. 1996; Lunau & Dyer 2024).

Modelling flower colours as perceived by bees is possible with colour vision models as, for example, the colour hexagon based on photoreceptor excitations as a generalized representation of colour opponency (Chittka 1992), in which the calculated excitation of the three photoreceptor types are plotted. In the colour space, the bee‐subjective hues are categorized according to the excitation of one or two photoreceptor types as UV, UV‐blue, blue, blue‐green, green, and purple (= UV‐green) (Fig. 4).

**Fig. 9:**
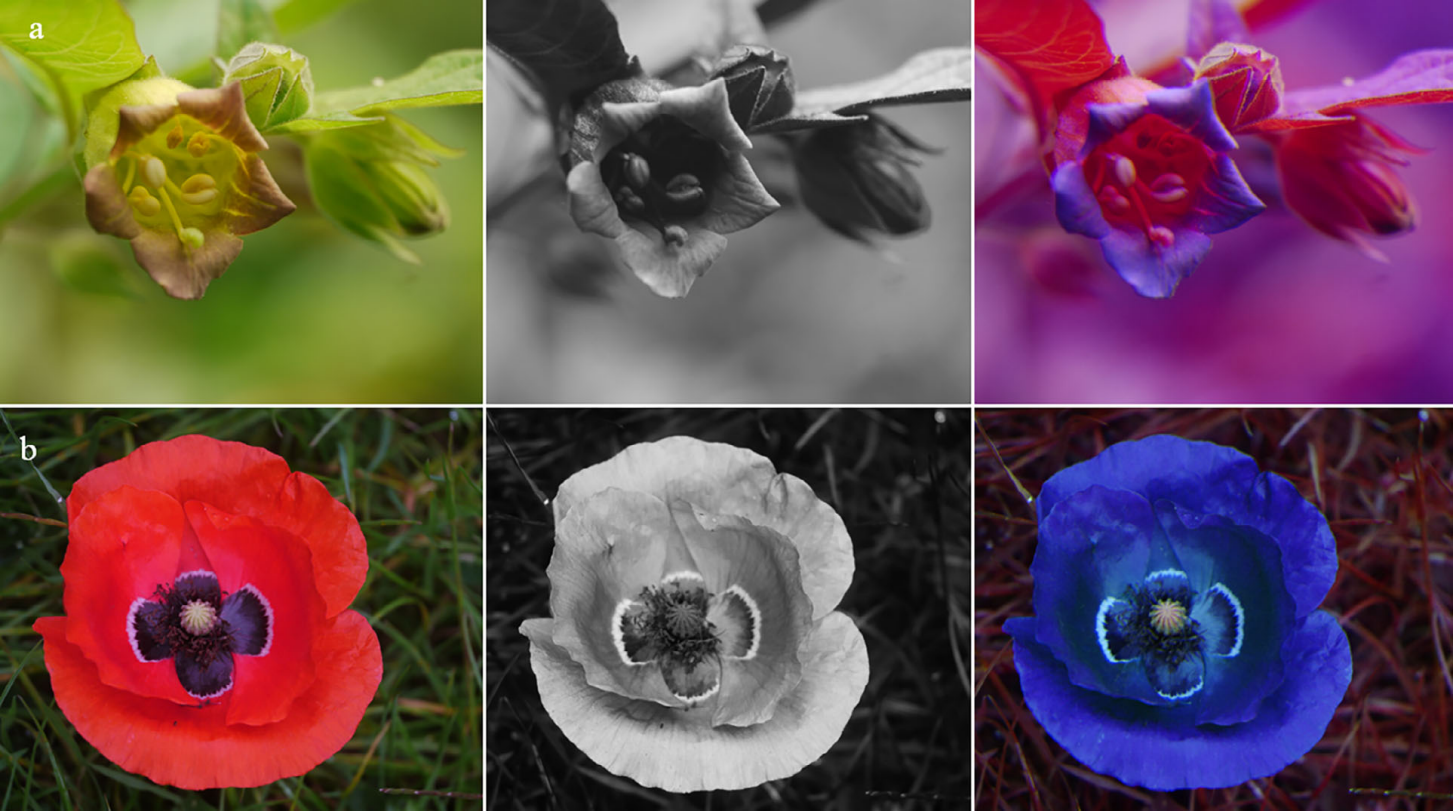
Colour photo, grey‐scale UV photo, and false colour photo in bee view of bee‐UV flowers (from left). (a) Human‐brown, bee‐UV flower of Deadly nightshade (*Atropa belladonna*) flowers and (b) human‐red, bee‐UV flower of Red poppy (*Papaver rhoeas*).

Bee‐UV flowers, that is, flowers which strongly excite bees' UV photoreceptors and thus are categorized as bee UV, are rare (Fig. 9). Red flowers are inconspicuous to red blind bees unless they also reflect UV light, as was shown by Lunau *et al*. (2011) when comparing the response of euglossine bees and hummingbirds to UV‐reflecting and UV‐absorbing red model flowers. The red poppy, *Papaver rhoeas*, represents a special case of a UV flower for bees. The red petals are UV‐absorbing in the Eastern Mediterranean, where they are pollinated by glaphyrid beetles, which are sensitive to red light and prefer red flowers as a rendezvous for mating. In the Western Mediterranean, native flowers of the same species become UV‐reflecting (Fig. 9b), where these beetles are absent, and the poppies switch to bee pollination (Martínez‐Harms *et al*. 2020). Some flowers that are dark grey or dark brown to our eyes, such as the Deadly nightshade (*Atropa belladonna*) strongly reflect UV light and are classified as bee‐UV (Fig. 9a).

## GLOSS AND UV

Glossy surfaces of flowers totally reflect light of all wavelengths. The glossy cuticle of smooth parts of flowers produces a dynamic visual signal, that is sparkle. Since the perception of glossy surfaces is dependent on the viewing angle and on the position of the sun, glossiness of flowers varies when a bee approaches them. Glossy floral structures thus display a non‐constant and thus little reliable visual cue for flower visitors.

**Fig. 10:**
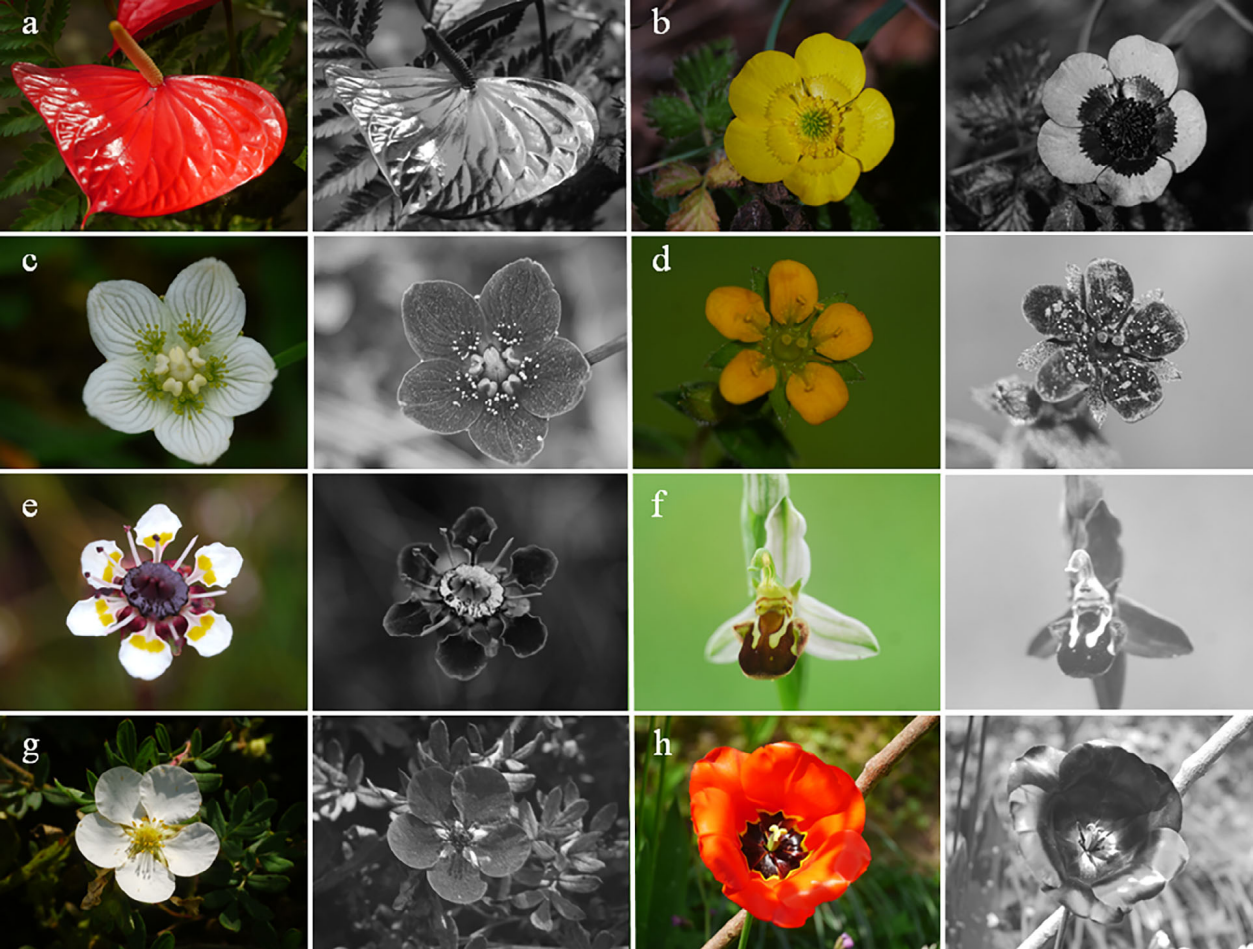
Colour photo (left) and UV photo (right) of gloss as visual floral cue in the flower of (a) *Anthurium* sp., (b) the floral guide of *Ranunculus* sp., (c) nectar mimicking staminode of *Parnassia palustris*, (d) nectar mimicking protuberances of *Saxifraga nigroglandulosa*, (e) nectar on the disc of *S. melanocentra*, and (f) on the labellum of the sexually deceptive orchid *Ophrys apifera*, (g) white petals and glossy sepals display different UV reflection in *Potentilla fructicosa* (white morph), (h) dark basal petal provides a contrasting background for glossy signals in *Tulipa* sp. (garden form).

There are a few instances in which flowers are glossy. As gloss originates from smooth surfaces, a co‐effect of glossiness is slipperiness, which means less grip for bees. In this context, flowers protect against nectar robbing bees by flat epidermal cells that appear glossy as a by‐product (Papiorek *et al*. 2014). Sexually deceptive flowers that mimic visual signals of female insects display glossy areas simulating the sparkle on the wings of the target females, as in *Ophrys* orchids (Vignolini *et al*. 2012; Fig. 10f). Alternatively, the sparkle is mimicked by white‐ and UV‐reflecting spots, as in the specialized beetle‐pollinated *Papaver rhoeas* (Fig. 9b; Lunau 2022) and in the ray florets of inflorescences of beefly‐pollinated *Gorteria* species (Johnson & Midgley 1997). In both cases the pollinators mate on the flowers and thus males in particular might be attracted by floral guides mimicking female conspecifics. As nectar is a glossy fluid reward in or on flowers, openly presented nectar and nectar‐mimicking structures on certain flowers are also glossy (Lunau *et al*. 2020; Wen *et al*. 2024; Fig. 10c–e). In white flowers, the reflection in the UV range of wavelengths represents an important difference between flower colour and glossy nectar (Fig. 10).

Non‐smooth surfaces can produce salient visual signals too. Conical epidermal cells reduce gloss, resemble velvet on flower petals and increase flower visibility to visitors by scattering light into a wide angular space (Stavenga *et al*. 2020). Nanostructures on petals with a distinct degree of disorder produce a halo of scattered light, predominantly at short wavelengths (ultraviolet and blue) that bees can use to discriminate flowers (Moyroud *et al*. 2017). Diffraction gratings on epidermal cells can cause iridescence in the visible and UV wavelength range (Whitney *et al*. 2009).

## FLOWER UV PATTERN AND UV RADIATION

UV, especially UV‐B is a significant environmental stressor for plants. The photons of UV light have greater energy than those of visible light; consequently, shortwave UV can cause radiation damage to DNA. Generally, high UV intensity reduces pollen viability in *Brassica rapa* (Gray *et al*. 2024). UV radiation causes damage to the DNA of pollen grains of alpine plants if the grains are exposed to UV radiation (Zhang *et al*. 2015). Therefore, UV absorption of pollen, anthers and parts of flowers that potentially reflect light onto pollen can protect pollen against damage from UV radiation (Koski *et al*. 2020; Fan *et al*. 2024; Lunau *et al*. 2024). By investigating and comparing of UV photos of the inner and outer side of petals of alpine flowers at high elevation, Fan *et al*. (2024) proposed that different colour patterns between inner and outer sides of flower petals may play a role in protecting pollen grains at the bud stage from UV radiation damage.

Therefore, aside from signalling to pollinators, UV absorption also has a protective function. The absorption of UV light by pollen grains, anthers and parts of the petals that might reflect light onto the anthers shields the DNA in the sperm of pollen grains against UV radiation (Koski *et al*. 2020; Fan *et al*. 2024). The diversity of floral colour patterns thus seems limited by the fact that flowers with open pollen presentation benefit from UV‐absorbing centre parts (Fig. 1). Flower with hidden stamens, for example papilionaceous flowers of the Fabaceae and Polygalaceae, might benefit from UV‐absorbing parts covering the anthers by limiting the transmission of UV light to the pollen (Lunau *et al*. 2024).

UV pigmentation could be a plastic and rapid evolving trait. Koski & Ashman (2015) examined patterns of variation in UV‐absorbing floral pigmentation in a widespread plant, *Potentilla anserina* (Rosaceae; Fig. 3). They found that the UV bull's eye increases with proximity to the equator in both hemispheres, and larger bull's eyes are associated with higher UV‐B incidence. Similarly, petals of *P. anserina* have larger UV‐absorbing petal areas at high elevations, where the up‐facing flowers are exposed to a higher and more variable UV radiation than at low elevations (Koski *et al*. 2022). Globally, regions with high UV radiation are high mountains and areas where ozone declines. Koski *et al*. (2020) found that pigmentation increased with ozone decline in taxa with pollen exposed to ambient UV radiation indicating such floral pigmentation change may impact plant pollination in future climate change scenarios. However, to our knowledge, until now, there are no studies at the community level investigating floral UV pattern to test if there are more flowers with UV patterning in high elevation or ozone decline habitats.

## CONCLUSIONS

In summary, our understanding of flower colours extends beyond mere visual appearance. Bees, with their distinct colour vision, likely interpret flower hues differently from humans. By appreciating this multifaceted perception, we gain deeper insights into the intricate relationship between pollinators and their preferred blossoms. UV light plays an important role for colour vision in bees and as a stressor for flower DNA. The absorption of UV light is typical for pollen and anthers shielding the DNA in the sperm of pollen grains against UV radiation. UV‐absorbing floral guides also prevent the reflection of UV light from petals to pollen. UV bee‐pollinated flowers are rare and either appear red or dull in colour to humans. The absorption of UV light is a necessary precondition for UV‐induced visible fluorescence, but the resulting fluorescence is a quantitatively neglectable visual phenomenon of flower coloration.

In the following we pick up the introductory questions and provide conclusions and elaborate the answers. Are there flowers that are bee‐UV (Shrestha *et al*. 2014)? Yes, bee‐UV flowers appear black, grey, brown, or red to humans and additionally reflect UV light. Typically, red bee‐pollinated flowers are bee‐UV due to reflection in the UV (Lunau *et al*. 2011). In contrast, red bird‐pollinated flowers are often UV‐absorbing (bee‐black) and discourage bees from visiting these flowers (Bergamo *et al*. 2016).

Are there flowers or parts of flowers that are invisible to bees (Chittka & Waser 1997)? No, all flower colours are visible to bees irrespective of its UV reflection or absorption. A noteworthy exception are translucent parts of trap flowers that exploit the positive phototropism of trapped bees and other insects (Faegri & Van der Pijl 1979).

How do bees see flowers lacking a UV pattern? If these flowers display a uniform colour, bees do not see any colour pattern. If these flowers display a pattern in the green or blue wavelength range, bees see a corresponding colour pattern. The absence of a UV pattern on yellow flowers is typical for bird‐pollinated flowers (Papiorek *et al*. 2016). Many flowers display the so‐called UV bull's eye (Lunau 2014). The term UV bull's eye was introduced for flowers that appear uniform in colour to humans but display differences in UV reflection. However, there are also flowers that display a blue bull's eye according to the limited range of wavelengths providing differences in reflection. Yellow flowers with orange floral guides might display a green bull's eye. In contrast to UV bull's eyes, these are visible to the human eye. However, even in UV bull's eyes of yellow flowers the pattern is often associated with slight differences in the visible wavelength range. How do bees respond to flowers lacking a UV pattern? Floral colour patterns, including UV patterns, are mostly associated with floral guides directing the bees towards the floral reward. Tiny flowers and flowers that are easy to handle may not need a floral guide to direct the bees towards to floral reward. In contrast to yellow bee‐pollinated flowers, yellow bird‐pollinated flowers lack UV pattern and thus might be difficult for bees to handle, and thus the lack of a UV pattern might discourage bees (Papiorek *et al*. 2016). For example, it has been shown that UV patternless flower morphs of *Anemone palmata* are less visited by bees and hoverflies (Rodríguez‐Castañeda *et al*. 2024).

How do bees perceive different features of flower colours, such as colour contrast, brightness, hue, and saturation? Green contrast, based exclusively on excitation of the green photoreceptor type, is essential to detect target flowers against the background when viewed at a small visual angle, that is, when the bee is still some distance away from the flower (Dyer *et al*. 2008; Van der Kooi & Kelber 2022). Colour contrast of the target flower against the background matters when the bee approaches the flower and passes over it at a species‐specific visual angle. Bees do not appear to use brightness cues in a colour processing context (Ng *et al*. 2018). Differences in hue are important for colour discrimination, whereas stronger saturation of flower colours is important for innate preferences (Rohde *et al*. 2013).

Is UV a specific floral signal? No, the colour vision system of bees is based on three equivalent types of photoreceptor, one of which is the UV receptor. However, there are visual tasks relying completely on the UV receptor, such as polarization vision, which is performed by a special dorsal rim area of the compound eyes lacking colour vision. Contrary to UV contrast, green contrast is perceived as a discrete signal by bees (Dyer *et al*. 2008) dependent on the visual angle of the target. Simply put, bees see a wildflower meadow colour‐blind with active green receptors but switch to colour vision when the visual angle of a target flower surpasses ca. 5° (Hempel de Ibarra *et al*. 2015; Fig. 2).

Does fluorescence contribute to flower colour? No, the UV‐induced visual fluorescence is too weak to significantly increase reflectance in the green and blue wavelength range (Lagorio *et al*. 2015).

Is UV an important component in glossy flower signals? Glossy structures in flowers totally reflect light and thus appear white to humans. By contrast, many white bee‐pollinated flowers absorb UV light and thus can be distinguished from glossy structures by bees. Glossy structures on flowers can provide important information for bees. Nectar is glossy, nectar‐mimicking structures may function as floral guides and thus also benefit from being glossy (Liao *et al*. 2020; Lunau *et al*. 2020; Wen *et al*. 2024). The glossy, nectar‐mimicking staminodes in *Parnassia wightiana* (Celastraceae) are attacked by florivorous beetles, thus influencing the pollinator attraction function of glossy staminodes (Wen *et al*. 2024).

Do flowers more often display UV‐absorbing parts in UV‐rich environments? Since UV radiation damages the DNA of pollen grains (Zhang *et al*. 2015), flowers benefit from UV absorption of pollen, anthers and parts of flowers that reflect light onto pollen (Koski *et al*. 2020; Lunau *et al*. 2024). Are distinct features of UV patterns associated with altitude and latitude? Yes, the size of the central UV‐absorbing parts on flowers increases with altitude (Koski & Ashman 2015; Koski *et al*. 2020). However, community‐level evidence is still lacking at high elevations and in ozone reduced environments.

## AUTHOR CONTRIBUTIONS

KL conceived the ideas; all authors wrote the manuscript and contributed to the drafts.
